# Nipah Virus Encephalitis Reemergence, Bangladesh

**DOI:** 10.3201/eid1012.040701

**Published:** 2004-12

**Authors:** Vincent P. Hsu, Mohammed Jahangir Hossain, Umesh D. Parashar, Mohammed Monsur Ali, Thomas G. Ksiazek, Ivan Kuzmin, Michael Niezgoda, Charles Rupprecht, Joseph Bresee, Robert F. Breiman

**Affiliations:** * Centers for Disease Control and Prevention, Atlanta, Georgia, USA;; † Centre for Health and Population Research, Dhaka, Bangladesh;; ‡ Office of Civil Surgeon, Naogaon, Bangladesh

## Abstract

We retrospectively investigated two outbreaks of encephalitis in Meherpur and Naogaon, Bangladesh, which occurred in 2001 and 2003. We collected serum samples from persons who were ill, their household contacts, randomly selected residents, hospital workers, and various animals. Cases were classified as laboratory confirmed or probable. We identified 13 cases (4 confirmed, 9 probable) in Meherpur; 7 were in persons in two households. Patients were more likely than nonpatients to have close contact with other patients or have contact with a sick cow. In Naogaon, we identified 12 cases (4 confirmed, 8 probable); 7 were in persons clustered in 2 households. Two *Pteropus* bats had antibodies for Nipah virus. Samples from hospital workers were negative for Nipah virus antibodies. These outbreaks, the first since 1999, suggest that transmission may occur through close contact with other patients or from exposure to a common source. Surveillance and enhancement of diagnostic capacity to detect Nipah virus infection are recommended.

## Abstract 2

Two Nipah virus encephalitis outbreaks in Bangladesh may be associated with person-to-person transmission.

Nipah virus is a recently described zoonotic paramyxovirus that causes a highly fatal encephalitis in humans (*1**,**2*). The only previously reported outbreaks of Nipah virus occurred in Malaysia and Singapore from September 1998 to May 1999. In Malaysia, 265 encephalitis cases, primarily among pig farmers, and a 40% death rate were reported (*3**–**5*). Concurrent outbreaks of a respiratory and neurologic illness caused by Nipah virus occurred among pigs in the affected areas, and close contact with pigs, especially sick pigs, was the major risk factor for human infection (*3**,**6*). Person-to-person transmission of Nipah virus was not documented (*7*). In Singapore, 11 cases and 1 death were reported among abattoir workers who slaughtered pigs imported from affected areas of Malaysia (*3**,**6*). The outbreak was contained by the mass culling of >1 million pigs, and since then, no other outbreaks of Nipah virus have been reported in Malaysia (*8*). Subsequent investigations identified *Pteropus* bats as a possible natural host for Nipah virus (*9**–**11*). *Pteropus* bats are also believed to be the natural host for Hendra virus, a zoonotic paramyxovirus that is genetically related to Nipah virus and has been associated with fatal respiratory and neurologic illness among persons in Australia (*12*).

In April and May 2001, a cluster of febrile neurologic illnesses with nine deaths was reported in a village in Meherpur District, Bangladesh. Preliminary investigations by the Bangladesh Ministry of Health and the World Health Organization (WHO) excluded a diagnosis of Japanese encephalitis, dengue fever, or malaria, but 2 of 42 serum specimens obtained from village residents in May 2001 showed reactive antibodies to Nipah virus antigen in tests performed at the U.S. Centers for Disease Control and Prevention (CDC). However, a comprehensive investigation of this outbreak was not conducted. In January 2003, a cluster of febrile illnesses with neurologic features and eight reported deaths occurred in adjoining villages in Naogaon District, ≈150 km from the village in Meherpur District. Similarities in the clinical manifestations observed among patients in Naogaon and Meherpur raised the question of whether the outbreaks were caused by the same agent.

In March 2003, we conducted a detailed retrospective investigation to describe the outbreaks in Meherpur and Naoganon, characterize their clinical features, and determine the etiologic agents, presence of asymptomatic infection, risk factors for infection and disease, and possible animal reservoirs.

## Methods

The field investigation took place March 6–16, 2003, and consisted of separate outbreak investigations in Meherpur and Naogaon districts, a cross-sectional study among healthcare workers in Meherpur Hospital, and an assessment of possible animal reservoirs in the outbreak regions. Because of the substantial time lapse between the outbreak period and the field investigation in Meherpur, approval for the Meherpur portion of the study was obtained from the appropriate ethical review committees. Informed consent was obtained from all participants, except for children <16 years of age, for whom consent was obtained from the parent or guardian. Approval for participation was obtained for children >7 years of age. In both outbreak investigations, the surveys were conducted among residents >2 years of age, which consisted of an oral interview and collection of 10 mL of blood by venipuncture. Field research assistants used a standardized data collection instrument to collect information on demographics, symptoms of illness, exposure to ill patients, exposure to animals in the surrounding area, and other possible risk factors. When persons were deceased, an interview was conducted by proxy with a household member. Interviews were typically completed within 30 minutes. We attempted to verify clinical information on hospitalized patients from medical records, but records were either not found or contained incomplete clinical data.

### Meherpur Outbreak Investigation

A population census was performed before the study by field research assistants, who visited each household and obtained information on the age and sex of each household member. Surveys took place in the villages of Chandpur (population 604), where persons who died or were hospitalized had resided, and Sishipara (population 237), an adjacent village located ≈1/2 km south of Chandpur. We defined the outbreak period as April 1 through May 31, 2001. Potential patients were identified from lists compiled by the initial WHO investigation and from self-reports of illness by village residents during the outbreak period. Household members were also surveyed as potential patients and assessed for clinically compatible illness or asymptomatic infection. To assess risk factors for infection, we enrolled controls by using simple random sampling of the numbered population census of remaining residents to select twice as many controls for each potential patient.

### Naogaon Outbreak Investigation

Surveys were conducted in the adjacent villages of East Chalksita (population 529) and Biljoania (population 481); suspected deaths and hospitalizations caused by Nipah virus infection were reported from both villages. The outbreak period was defined as January 1–31, 2003. Because the scope of the outbreak was not well-defined, emphasis was given to case finding, which consisted of a household-to-household search by the field research assistants to identify potential cases. A sample of asymptomatic household members and other village residents, selected by simple random sampling from an available government population census, were surveyed for illness and serologic evidence of infection.

### Healthcare Worker Study

A cross-sectional survey was performed at Meherpur District Hospital, where most of the ill residents from Chandpur were admitted with encephalitis symptoms. We interviewed and collected serum samples from healthcare workers whose job descriptions involved close contact with patients, such as physicians, nurses, orderlies, and nursing assistants. Information was obtained on demographics, symptoms of illness, the degree of contact with the patient, and type of barrier precaution used during patient care.

### Serum Sample Collection

Blood specimens were centrifuged on site, transported on wet ice, and stored at –20°C. Serum samples were shipped frozen at –70°C to CDC and tested with an immunoglobulin (Ig) M capture enzyme immunoassay (EIA) for detection of Nipah/Hendra IgM antibodies and an indirect EIA for Nipah/Hendra IgG antibodies (*13*). Nipah (Malaysia prototype) virus antigen was used in both assays.

### Data Analysis

Interview data were entered into Epi Info 6.04 (CDC, Atlanta, GA) and validated and analyzed by using SAS version 9 (SAS, Cary, NC). Based on serologic results, we defined a confirmed case as a case in a village resident with fever, headache, or altered level of consciousness within the specific outbreak period with antibodies reactive with Nipah antigen. A probable case was defined as a case in a resident with onset of fever plus headache or altered level of consciousness during the outbreak period who died before serum samples could be collected for testing. For the case-control study, univariate analysis was performed for each risk factor variable. Potential cases, including those in household members who did not have cases that met definition for confirmed or probable cases, were defined as noncases and analyzed together with the control group.

### Assessment of Animal Reservoirs

In both districts, attempts were made to obtain representative samples from domestic and wild birds and mammals. Collections were based in part on relative abundance, suggestions of any history of ill animals indicated by village reports, and the likelihood for human contact. Domestic species were restrained manually, when possible, or sedated by the intramuscular administration of ketamine hydrochloride (≈5–10 mg/kg). Traps were set near village residences for small mammals, such as rodents and insectivores. Mist netting or hand collections for bats and birds occurred in and around homes, suspected flyways, roosts, such as abandoned buildings, and likely foraging areas, such as fruit plantations. In addition, samples were obtained from bats captured or killed by local villagers because they were suspected of feeding on fruit trees in nearby orchards. In Naogaon, villagers reported that a herd of pigs was in the vicinity of the village ≈2 weeks before the outbreak. Pigs owned by the same herder (but not from the same herd) were bled for serologic testing. Animal serum samples and tissue were shipped frozen to CDC, and all specimens were tested using an indirect EIA employing protein A/G conjugate for mammals and an antibird conjugate for avian species.

## Results

### Meherpur Outbreak Investigation

In Meherpur, 13 residents, all from Chandpur (attack rate 2.1%), met the case definition: 4 had confirmed cases and 9 had probable cases. The outbreak, which spanned 1 month, began with the index patient, a 33-year-old farmer who had onset of symptoms on April 20, 2001 and died 6 days later. The outbreak ended with the last case which occurred in a 60-year-old woman, a neighbor of the index patient, with onset of symptoms on May 20 (Figure A). All nine persons with probable cases were hospitalized and died as a result of their illness before laboratory specimens could be collected (case-fatality rate = 69%). The average length of illness from onset to death was 6 days (range 3–10 days). All four persons with confirmed cases had IgG antibodies reactive to Nipah virus antigen (including two persons that had previous positive results from initial testing in May 2001). IgM antibodies were not detected in any of the specimens. Six (46%) of 13 patients were male; their ages were 4–60 years of age (median 38 years). A cluster of five cases occurred in persons from the same household as the index patient; the cluster consisted of the index patient's wife, son, brother, and sister. Eight separate households were affected, and 9 of the 13 persons with probable or confirmed cases were relatives of the index patient either by blood or marriage. Although patients with probable or confirmed cases lived in the western half of the village, no other obvious geographic clustering was noted; households with no cases were located in between those with cases.

**Figure:**
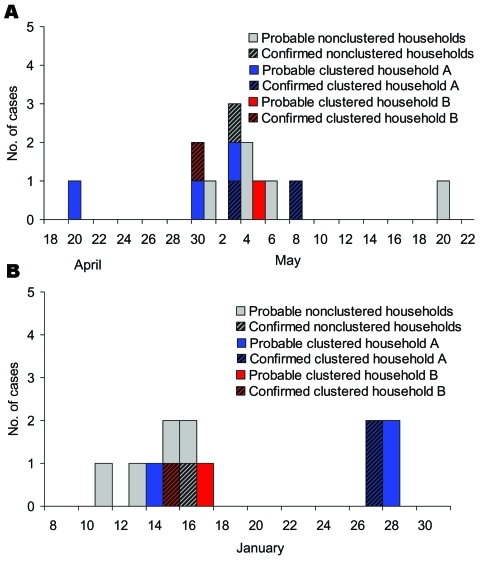
Illness onset of probable and confirmed cases of encephalitis. A) Meherpur District, 2001. B) Naogaon District, 2003.

Of 119 surveys completed in Meherpur, 96 (81%) were from Chandpur residents. Of these, 15 (16%) were initially identified as potential cases, 28 (29%) were their household members, and the remaining 53 (55%) were randomly selected as controls. All the patients came from the group identified as persons with potential cases in Chandpur; no patients were found among their household members or the randomly selected controls. The case-control analysis was restricted to Chandpur residents. With respect to baseline characteristics, patients (n = 13) did not differ from nonpatients (n = 83) by sex or occupation, but patients were older than nonpatients (mean age 40 vs. 27 years of age, p < 0.001). We examined close contact with patients and contact with animals as potential risk factors for illness. Persons who lived with or cared for patients during the time of their illness were more likely to become patients themselves (odds ratio [OR] 4.80, 95% confidence interval [CI] 1.23–18.8) (Table 1). In a subanalysis among those who lived with or cared for patients, no significant difference was found between patients and nonpatients with regard to sharing items of a personal nature, such as toothbrush or utensils, but patients were more likely to have touched secretions, such as urine or saliva, of other patients (OR 5.7, CI 1.0–32.7). Among all interviewed residents, patients were more likely than nonpatients to have had contact with an ill cow (OR 7.9, CI 2.2–27.7). Although >90% of villagers reported that bats were frequently seen near their homes, patients and controls showed no differences in contact with bats or other animals, whether ill or well.

**Table 1:** Risk factors for illness among patients and nonpatients, Chandpur village, Meherpur, Bangladesh, 2001

Characteristic	Patients	Nonpatients	OR (95% CI)^a^
Caring for or living with a person with a case	10/13	34/83	4·80 (1.23–18.8)
Shared personal items	7/10	24/29	0·49 (0.09–2·56)
Contact with secretions from a person with a case	7/9	11/29	5·73 (1.00–32·7)
Any animal contact			
Chickens	13/13	83/83	Undefined
Cows	13/13	68/83	6.11 (0.34–108.4)
Dogs	12/13	78/83	0.76 (0.08–7.16)
Goats	10/13	64/83	0.98 (0.25–3.97)
Ducks	9/13	58/83	0.97 (0.27–3.45)
Bats	3/13	36/83	0.39 (0.10–1.53)
Sick animal contact			
Chicken	7/13	44/ 83	1.03 (0.32–3.34)
Cow	8/13	14/83	7.89 (2.24–27.7)
Dog	0/13	2/83	1.20 (0.05–26.5)
Goat	5/13	23/83	1.63 (0.48–5.50)
Duck	1/13	24/83	0.20 (0.03–1.66)

^a^OR, odds ratio; CI, confidence interval.

### Naogaon Outbreak Investigation

In Naogaon, 12 persons from both villages met the case definition (attack rate 1.1%): 4 cases were confirmed, and 8 were probable. All cases were found through the reports of initial deaths and hospitalizations; no patients were found among asymptomatic family members or randomly sampled village residents. The index case occurred in a 12-year-old boy with symptom onset on January 11, 2003, and the last case occurred in 12-year-old girl on January 28 (Figure B). All but one of the patients were hospitalized (one died before hospital admission), and all eight patients with probable cases died (case-fatality rate = 67%); mean period from onset of symptoms to death was 4 days (range 2–7 days). No diagnostic specimens were available from the deceased patients. Of the four confirmed patients, all had IgG antibodies and three had IgM antibodies reactive with Nipah virus antigen. Eight (67%) patients were male, and their ages were 4–42 years of age (median 12 years of age). Clustering of patients occurred in one household, in which the head of household became ill on January 14 and later died. Symptoms developed in his wife and three daughters 2 weeks later; only the two younger girls survived. Altogether, eight households were affected. As with Meherpur, no geographic clustering of affected households was seen, but in contrast to the outbreak in Meherpur, members of affected households had no blood relationship to those in other affected households.

### Clinical Features of Cases

The typical clinical course was similar in both outbreaks and began with onset of fever, followed by headache and varying degrees of diminishing consciousness. In both outbreaks, a fever was found in all patients, followed by an altered level of consciousness in 22 (88%) and headache in 18 (72%) (Table 2). Cough (16 [65%]) and difficulty breathing (16 [65%]) were also common. Vomiting occurred in half of the patients, but seizures and diarrhea were uncommon. Although a significant difference was seen between patients with confirmed and probable cases only with regard to dyspnea (25% vs. 82%, p = 0.01), patients with probable cases (all of whom died) tended to have a higher proportion of all symptoms compared to patients with confirmed cases, all of whom survived.

**Table 2:** Clinical characteristics of patients with probable and confirmed encephalitis cases, by case classification, Meherpur and Naogaon, Bangladesh, 2001 and 2003

Symptom	Patients with			
	Confirmed cases, n (%)	Probable cases, n (%)	p value	Total patients n (%)
Fever	8 (100)	17 (100)	1.00	25 (100)
Altered level of consciousness	6 (75)	16 (94)	0.17	22 (88)
Headache	4 (50)	14 (82)	0.12	18 (72)
Cough	3 (38)	13 (76)	0.08	16 (64)
Dyspnea	2 (25)	14 (82)	0.01	16 (64)
Vomiting	4 (50)	9 (53)	0.61	13 (52)
Seizures	1 (13)	5 (29)	0.34	6 (24)
Diarrhea	0 (0)	3 (18)	0.30	3 (12)

### Healthcare Workers Study

A total of 46 healthcare workers (6 physicians, 20 nurses, 20 ward assistants) participated in the survey; 32 (70%) reported having contact with at least one of the encephalitis patients through the course of direct patient care. Of those who had direct patient contact, 12 (40%) used barrier precautions, such as gloves, masks, or gowns. One worker had unprotected mucous membrane contact with secretions of ill patients with encephalitis, and one reported a needlestick injury. Six workers reported an illness characterized by fever and headache in the period from the outbreak onset through June 30, 2001, but none reported mental status changes. None of the participating healthcare workers had antibodies reactive with Nipah virus antigens.

### Assessment of Animal Reservoirs

No cluster of ill animals was observed or reported in either district. In Meherpur, serum samples were collected from two pigs and 31 bats, including 25 *P. giganteus*. None had antibodies reactive with Nipah virus antigens. In Naogaon, 50 animals were tested for evidence of Nipah-like virus infection: 10 birds, 4 pigs, 4 dogs, 2 shrews, 5 rodents, and 25 bats, including 19 *P. giganteus*. Antibodies reactive to Nipah virus antigen were detected in two *P. giganteus* adult females. Serum specimens from all other animals were negative.

## Discussion

In these two outbreaks, antibodies reactive with Nipah virus antigen were found in seriously ill persons with encephalitis and antibodies were absent in asymptomatic persons or those without serious illness. These findings strongly suggest that Nipah, or a related virus, is the cause of both outbreaks. Nipah virus–associated illness has not been previously reported outside of Malaysia and Singapore. However, in contrast to the outbreaks in Malaysia, where animal illnesses were reported and close contact with pigs was strongly associated with Nipah virus infection in Bangladesh, no obvious zoonotic source has been identified. Pigs are infrequently found in Bangladesh, and no animal illnesses or die-offs in or around the affected villages were reported. Although case-control results indicated that patients were more likely to have contact with an ill cow, no such cow was available for testing, and the associations may have been due to chance. However, such potential risk factors need to be explored in future outbreak settings. Because antibodies reactive with Nipah virus were identified in local *Pteropus* bats, which reinforces previous findings, this genus may serve as the reservoir for this group of viruses (*9**–**11*). A possible explanation for acquisition of infection without an obvious domestic reservoir may be inadvertent direct contact with bats or bat secretions.

Human-to-human transmission of Nipah virus was not shown in the Malaysia and Singapore outbreaks (*7*), but several findings from the Bangladesh outbreaks suggest that close contact may have resulted in transmission. In Meherpur and in Naogaon, clusters of cases occurred within family households, with dates of symptom onset occurring over a range of time. In Meherpur, relatives with close contact with patients became ill, and handling or exposure to secretions of patients was found to be a risk factor for illness. Nipah virus has been detected in respiratory secretions and urine of patients, which suggests that person-to-person transmission is possible (*14*). However, we cannot rule out the possibility that a common source within households and among relatives may have been responsible for infection. In contrast, we found no evidence for transmission of Nipah virus from patients to healthcare workers. Contact between secretions or blood of healthcare workers and patients was reported in only two instances, which is an insufficient number to assess transmissibility through these routes. However, the lack of symptoms and lack of detectable antibody to Nipah virus in all hospital staff we evaluated suggest that transmission from patients to healthcare workers is uncommon.

The major clinical characteristics described in Bangladesh were generally similar to the characteristics described during the Nipah virus outbreaks in Malaysia and Singapore, with most persons having fever, headache, and an altered level of consciousness (*2*). In Bangladesh, a higher proportion of patients had an altered level of consciousness than those in Malaysia, although the results in our study relied on self-reporting, and objective descriptions of symptoms were not systematically documented. The absence of antibodies to Nipah/Hendra virus in asymptomatic persons suggests that subclinical infection did not occur or was an uncommon event, although subclinical infection has been previously documented (*15**,**16*). Among patients with probable or confirmed cases, patients in Naogaon tended to be younger (median age 12 years vs. 38 years of age) and to have a shorter interval from symptom onset to death (4 days vs. 6 days), compared to patients in the outbreak in Meherpur. Whether younger age is associated with a more fulminant course is uncertain, but the experience in Naogaon suggests that children appear to be as susceptible to infection as adults.

We restricted our case definition to confirmed or probable cases and did not include suspected cases in classification, defined as a surviving resident with onset of fever plus headache or altered level of consciousness, without serologic evidence for Nipah virus infection. In Meherpur, five nonpatients would have had suspected cases; in Naogaon, 44 patients would have had suspected cases. In the absence of objective clinical and serologic findings, persons with suspected cases are more likely to be patients with false-positive test results. On the other hand, the sensitivity of the Nipah virus EIA is limited (*13*); determining whether suspected cases represented true Nipah virus infection versus another process (such as hysteria or a different clinical syndrome) is difficult. Another limitation, especially of the Meherpur investigation, was the difficulty of obtaining an accurate recollection of activities that took place almost 2 years before the study.

Two features of this outbreak of Nipah virus encephalitis are distinct from previous outbreaks. A clear history of exposure to a specific species of animals was lacking, although bats in the region had serologic evidence of infection, and person-to-person spread may have been an important mode of transmission. Two independent clusters of cases suggest that this virus may sporadically infect humans. From January through April 2004, two new clusters of fatal Nipah virus encephalitis have been reported in Bangladesh. These outbreaks further underscore the need for enhancing regional surveillance for Nipah virus and clarifying transmission patterns. Also, increasing the capacity to conduct surveillance for new cases may add to our understanding of the disease and guide development of effective prevention strategies.
